# A review of mathematical models of human trust in automation

**DOI:** 10.3389/fnrgo.2023.1171403

**Published:** 2023-06-13

**Authors:** Lucero Rodriguez Rodriguez, Carlos E. Bustamante Orellana, Erin K. Chiou, Lixiao Huang, Nancy Cooke, Yun Kang

**Affiliations:** ^1^Simon A. Levin Mathematical and Computational Modeling Sciences Center, Arizona State University, Tempe, AZ, United States; ^2^Human Systems Engineering, Arizona State University, Mesa, AZ, United States; ^3^Center for Human, Artificial Intelligence, and Robot Teaming, Global Security Initiative, Arizona State University, Mesa, AZ, United States; ^4^Sciences and Mathematics Faculty, College of Integrative Sciences and Arts, Arizona State University, Mesa, AZ, United States

**Keywords:** trust, mathematical modeling, dynamical models, human-autonomy teaming, reliance, decision-making, risk dynamics, trust measures

## Abstract

Understanding how people trust autonomous systems is crucial to achieving better performance and safety in human-autonomy teaming. Trust in automation is a rich and complex process that has given rise to numerous measures and approaches aimed at comprehending and examining it. Although researchers have been developing models for understanding the dynamics of trust in automation for several decades, these models are primarily conceptual and often involve components that are difficult to measure. Mathematical models have emerged as powerful tools for gaining insightful knowledge about the dynamic processes of trust in automation. This paper provides an overview of various mathematical modeling approaches, their limitations, feasibility, and generalizability for trust dynamics in human-automation interaction contexts. Furthermore, this study proposes a novel and dynamic approach to model trust in automation, emphasizing the importance of incorporating different timescales into measurable components. Due to the complex nature of trust in automation, it is also suggested to combine machine learning and dynamic modeling approaches, as well as incorporating physiological data.

## 1. Introduction

Rapid advances in automation technologies, including autonomous vehicles, robotics, autonomous web-based systems, and user experience frameworks and decision aids, are dramatically impacting almost every aspect of our daily life. Understanding how humans work with automation is vital for automation to work most beneficially for humans. *Trust*, which is not always consistently defined and operationalized, has been identified as a key factor influencing human-automation interactions (Lee and Moray, 1992; Muir, 1994; Lee and See, 2004; Kohn et al., 2021).

Given the importance of trust in human-automation interactions, it is critical to understand the different definitions and operationalizations of trust in the literature. Some researchers define trust using an interpersonal framing, such as the *“expectancy held by an individual that the word, promise or written communication of another can be relied upon”* (Rotter, 1967). Subsequently, Mayer et al. (1995, p. 712) defined trust as the *“willingness of a party to be vulnerable to the actions of another party based on the expectation that the other will perform a particular action important to the trustor, irrespective of the ability to monitor or control that party.”* This definition identifies that individuals must willingly put themselves at risk. Similarly, Kramer (1999, p. 571) defined trust as a behavioral result or state of vulnerability, such as *“a state of perceived vulnerability or risk that is derived from an individual's uncertainty regarding the motives, intentions, and perspective actions of others on whom they depend.”* Falcone and Castelfranchi (2001) defined trust as a mental state, a belief of a cognitive agent to achieve a desired goal through another agent. Based on previous work by Fishbein and Ajzen (1975), and Lee and See (2004, p. 54) described reliance as a behavior that can indicate trust and stated that trust itself is *“the attitude that an agent will help achieve an individual's goals in a situation characterized by uncertainty and vulnerability.”* This is a widely used definition that integrates across several disciplines and is careful to point out that conflating trust with intent, behaviors, or beliefs could be misleading for future research (Lee and See, 2004).

To further understand the concept of trust in human-automation interactions, it is important to examine the different sources of variability in trust. Hoff and Bashir (2015) reviewed the empirical work that followed Lee and See (2004) and defined three sources of variability in trust in automation: dispositional, situational, and learned. Dispositional trust is a person's attitude toward autonomous agents based on pre-existing knowledge and demographic characteristics. Dispositional trust precedes interaction with a specific agent; it is based on individuals' own personalities, beliefs, and proclivities. Learned trust is based on prior experience with a specific autonomous system. Through this experience, operators learn about the system's capabilities, observe its performance, and develop expectations about the system's reliability. Learned trust is impacted by performance; for example, operators judge automation that fails on easy tasks more harshly (greater trust degradation) than agents that fail on difficult tasks (Madhavan et al., 2006). Situational trust considers conditions in the environment. This may include the external context, for example, the impact of weather on task difficulty; it may also include internal variables, such as the operator's current mental load or emotional state. The same system that is trusted in one situational context may not be trusted in a different context. Situational variables may change quickly and be difficult to observe, adding complexity to any effort to assess trust. Learned trust can change as an operator gains new experience with an agent and it may shift if an agent suddenly demonstrates a behavior change or experiences a failure. Situational trust can be unstable; anything altering the internal or external environment may impact the operator's moment-to-moment trust in an agent–even factors that are not directly related to the agent or its performance. The categorization of dispositional trust, learned trust, and situational trust additionally suggests distinguishable time frames at which to capture trust and its associated outcomes, at various levels of a system (i.e., from micro to macro-level outcomes).

Understanding the various sources of variability in trust is crucial for comprehending the relationship between trust and reliance in human-automation interactions. When choosing whether to rely on an autonomous agent, trust (an internal state) precedes reliance (an observable behavior). Reliance (choosing to use the autonomous agent with some risk involved) is a behavioral proxy measure for trust. The two are not perfectly related, although they do generally correlate positively. This relationship has been demonstrated to increase in strength with the complexity of combat autonomy and novelty of situations (e.g., Sanders et al., 2019). Like trust, decisions to rely on another entity can change over time, as a situation evolves. However, although trust is often examined as it coincides with observable behaviors like reliance, the strength of the relationship between an operator's trust ratings (their reflective responses) and behavior (how the operators act in the moment) can vary widely. Literature has found that automation performance, workload, environment, and risk largely affect decisions to use automation (Parasuraman and Riley, 1997; Lee and See, 2004; Lee, 2008; Kohn et al., 2021). Psycho-social models such as the Technology Acceptance Model (TAM; Davis, 1989) and the Unified Theory of Acceptance and Use of Technology (UTAUT; Venkatesh et al., 2003) also suggest that factors equivalent to automation performance and workload influence intentions, and subsequently influence behavior (reliance).

Given the challenges associated with relying solely on self-report measures and observable behaviors to assess trust in automation, the use of physiological measures such as electroencephalography (EEG) and Galvanic skin response (GSR), gaze behavior, and electrocardiogram (ECG) may serve as promising real-time indicators that reflect trust dynamics when interacting with automation. Trust has been measured by surveys, binary behavioral indicators, sensory-based physiological values, and communication patterns (Huang et al., 2020, 2021). Binary behavioral indicators of trust include use or non-use of the automation and duration of use, as well as eye-tracking fixation duration (Jenkins and Jiang, 2010; Gremillion et al., 2016). Sensor-based psychological measures, such as EEG and GSR, are more nuanced than surveys and binary behavioral indicators for tracking and deciphering trust dynamics. Recorded physiological data during human-automation interaction is typically analyzed through the tools of statistical analysis and machine learning (Gremillion et al., 2019; Walker et al., 2019; Neubauer et al., 2020; Oh et al., 2020; He et al., 2022). Although using physiological measures is not without its challenges largely because their interpretation depends highly on specific task contexts, and therefore still requires either time-intensive manual annotation or highly controlled task environments that are difficult to generalize, there remains a demand for real-time understanding and indicators of trust in automation during operation. This suggests that developing mathematical models of trust in automation that incorporate both behavioral and physiological data would be highly valuable.

As the demand for real-time indicators of trust in automation grows, there is a need for more comprehensive trust models. Trust definitions and related concepts are crucial for developing dynamical models to better understand trust in automation dynamics. Over the past few decades, researchers have been actively working on models to examine the dynamics of trust and reliance on automation. Kok and Soh (2020) performed a comprehensive narrative review that addresses known methods used to capture trust in automation and proposes future directions for research. The authors suggest the need for trust measures that are both lightweight and effective across various levels of automation, embodiment, and mental perception. They argue that several popular trust models lack empirical support and do not easily correspond to existing measures of trust. As a solution, the authors propose a model based on the measurable components of trust, which could be defined in detail and serve as a simplified framework for discussing and categorizing trust measures. More recent theoretical work on trust (Chiou and Lee, 2021) has expanded from the uni-directional information processing model of trust (and reliance) to discuss a more bi-lateral relational approach to trust (and cooperation). However, this theory remains conceptual and requires the specification of the goal environment, which varies widely, at least compared to task contexts that fit neatly within signal detection theory. Moreover, few empirical papers have yet attempted to develop models based on this relational concept of trust. In this paper, we revisit previous work on the well-studied information processing perspective of trust and reliance. We aim to provide the scientific community with an overview, from the mathematical and human factors perspective, of different mathematical modeling approaches, their limitations, feasibility, and generalizability for trust dynamics in various human-automation interaction contexts. We also emphasize the importance of incorporating different timescales into measurable components when modeling trust in automation. Additionally, we propose a framework and provide an example to model trust in automation through dynamic measures that capture the variability of the environment.

## 2. Mathematical Models of Trust in Automation

In this section, we will review mathematical models for trust in automation using a discrete-time modeling approach, including the application of decision field theory, step-function approach, and probabilistic approach. In the following subsection, we review mathematical models that have some component of randomness or unpredictability in their structure.

### 2.1. Stochastic Difference Equations

Rempel et al. (1985) proposed that trust is a dynamic attitude that follows a particular sequence of dimensions to form gradually over time. He also identified predictability, dependability, and faith as the three dimensions that influence an individual's acceptance of a trustee to form the basis of trust. This concept was subsequently applied to mathematical models of trust in automation by Lee and Moray (1992, 1994), Muir (1994), Muir and Moray (1996), and Lee and See (2004), as described below.

#### 2.1.1. Lee and Moray Models

Lee and Moray (1992) used linear regression models to examine the factors affecting trust in automation and used dynamical models to explore how trust changes over time. Their model for trust dynamics was validated using data from a laboratory experiment of participants operating a simulated orange juice pasteurization plant. In this experiment, each participant reported their trust level (on a scale from 1 to 10) at the end of each of 60 trials recorded over 3 days.

Lee and Moray (1994) started with the development of linear regression models for factors affecting trust. The fitting of these linear regression models suggested that self-reported trust was influenced by two factors, namely, the occurrence of a fault in a system and the performance of the system as measured by the total output efficiency (total output/total input). Notably, the linear regression models proposed in Lee and Moray (1994) did not reflect the dynamic response of trust to these variables. Instead, they predicted trust as a linear combination of the current level of performance and the fault in the system. However, the model did not consider past occurrences of faults, past values of performance, past values of trust, or the relationship between past and current values due to the possible changes in the work environment.

To give information about the memory of trust, and/or the effect of past occurrences of faults and performance, Lee and Moray (1992, 1994) proposed and used a stochastic difference model for trust *T*(*t*), which depends on the performance *P*(*t*) and fault *F*(*t*) of the automation in the current trial *t* and previous trial at *t* − 1.


(1) $$\begin{array}{l} T\left(t\right)=\phi_{1}T\left(t-1\right)+\underset{\text{Performance}}{\underset{︸}{A_{1}P\left(t\right)+A_{1}\phi_{2}P\left(t-1\right)}}\\ \;+\underset{\text{Occurrence of faults}}{\underset{︸}{A_{2}F\left(t\right)+A_{2}\phi_{3}F\left(t-1\right)}}+a\left(t\right), \end{array}$$

where *A*_1_ is the weighting of system performance, *A*_2_ is the weighting of the occurrence of a fault, ϕ_1_ and ϕ_2_ are time constants of the Autoregressive Moving Average Vector (ARMAV) model, and *a*(*t*) is a random noise perturbation at trial *t*. The work of Lee and Moray (1992) provides a first step toward modeling trust between humans and machines and its influence on operators' control strategies and decision-making. Trust model (1) assumes that trust dynamics at trial *t* is a linear combination of (1) trust *T* at the previous trial (*t* − 1); (2) performance *P* of the system at trial *t* and *t* − 1; (3) the occurrence of a fault *F* at trial *t* and *t* − 1; and (4) environment perturbations *a*(*t*) that are beyond the operator's control. Items (2) and (3) can be linked to the risk assessment of using automation when the participant makes a decision to use automation or not.

One limitation of model (1) is that it does not explain and test why trust is a linear combination of previous trust, performance, and fault. The application of trust model (1) may be too restricted, as it can only be applied to the specifically mentioned experiment since it requires the input of performance and faults. To have a more general model, there is a need to define performance, faults, and other influencing factors (e.g., workload and risk), and the relationships between these variables.

#### 2.1.2. Muir and Moray Models

Muir (1994) adopted the trust definition from Barber (1983) and formulated the following definition of trust in automation:

"*Trust (T) is the expectation (E), held by a member of a system (i), of persistence (P) of the natural (n) and moral social (m) orders, and of technically competent performance (TCP), and of fiduciary responsibility (FR), from a member (j) of the system, and is related to, but is not necessarily isomorphic with, objective measures of these properties*." (p. 1,911)

This definition can be summarized as the equation below:


(2) $$\begin{array}{r} T_{i,j}=\underset{\text{Expectation of persistence}}{\underset{︸}{E_{i}\left(P\left(n,m\right)\right)}}+\underset{\begin{array}{c} \text{Expectation of technically}\\ \text{competent performance} \end{array}}{\underset{︸}{E_{i}\left({\text{TCP}}_{j}\right)}}\\ +\underset{\begin{array}{c} \text{Expectation of fiduciary}\\ \text{responsibility} \end{array}}{\underset{︸}{E_{i}\left({\text{FR}}_{j}\right)}}, \end{array}$$

where *T* is a composite expectation, comprised of three expectations: *P*, which is the fundamental expectation of persistence; TCP, which includes skill-, rule-, and knowledge-based behaviors; and FR includes notions of intent, power, and authority. The trust model (2) suggests simple linear impacts from persistence (*P*), technically competent performance (TCP), and fiduciary responsibility (FR), when in fact a multiplicative model or a more complex model may turn out to be a more accurate mathematical representation. For example, the three component expectations need not be equally important; each component expectation may have to be weighted according to its importance in a particular context and may have nonlinear impacts. Muir proposed the following hypothetical regression model of human trust in a human or machine referent,


(3) $$\begin{array}{l} T_{i,j}=B_{0}+B_{1}E_{i}\left(P\left(n,m\right)\right)+B_{2}E_{i}\left({\text{TCP}}_{j}\right)+B_{3}E_{i}\left({\text{FR}}_{j}\right)\\ \;+B_{4}E_{i}\left(P\left(n,m\right)\right)E_{i}\left({\text{TCP}}_{j}\right)\\ \;+B_{5}E_{i}\left(P\left(n,m\right)\right)E_{i}\left({\text{FR}}_{j}\right)+B_{6}E_{i}\left({\text{TCP}}_{j}\right)E_{i}\left({\text{FR}}_{j}\right)\\ \;+B_{7}E_{i}\left(P\left(n,m\right)\right)E_{i}\left({\text{TCP}}_{j}\right)E_{i}\left({\text{FR}}_{j}\right), \end{array}$$

where *B*_0−7_ are parameters. According to Muir's (1994) model (2), based on the Rempel et al. (1985) stage model, Muir and Moray (1996) showed that predictability should be the best predictor of overall trust early in an operator's experience, followed later by dependability and then faith. Thus, they proposed model (4) that was applied to their experiment data. This model was then extended to model (5) by including three additional components.


(4) $$\begin{array}{l} \text{Trust}=\text{Predictability}+\text{Dependability}+\text{Faith} \end{array}$$

(5) $$\begin{array}{l} \text{Trust}=\text{Predictability}+\text{Dependability}\\ \;+\text{Faith}+\text{Competence}+\text{Responsibility}+\text{Reliability} \end{array}$$

Muir's models (2) and (5) are extended from Barber's (1983) and Rempel et al.'s (1985) models and showed that the perceived predictability is one of the bases of trust, which, in turn, is the foundation for an operator to estimate the future behavior of a referent. The accuracy of that prediction may be assessed by comparing it with the actual behavioral outcome. Besides, a person who makes an estimate may associate a particular level of confidence with such an estimate. Hence, confidence is a qualifier related to a particular estimate. Confidence is not synonymous with trust. An important limitation is how to operationalize and measure those three components: predictability, dependability, and faith.

#### 2.1.3. Busemeyer and Townsend Model

Decision Field Theory (DFT) is a dynamic-cognitive approach to human decision-making based on principles psychological rather than economic ones (Busemeyer and Diederich, 2002). This type of model has been used to understand the evolution of the preferences among options of a human decision-maker (Lee et al., 2008). DFT provides a mathematical approach to understanding the cognitive and motivational mechanisms that guide people in the process of decision-making within a changing environment. Busemeyer and Townsend (1993) applied DFT to the decision-making process of automation use or disuse, which involves two options: relying on automation (A) or using manual control (M). Let *S*_1_ and *S*_2_ correspond to uncertain events, where *S*_1_ is the occurrence of an automation fault and *S*_2_ is the occurrence of a fault that compromises manual control. Variables *y*_*Mj*_ and *y*_*Aj*_ are the possible payoffs if event *S*_*j*_ occurs, where *j* = 1, 2. This model has two basic Subjective Expected Utility (SEU) functions given by:


$$\begin{array}{l} \;V_{A}\left(n\right)=W\left(S_{1}\right)u\left(y_{A1}\right)+W\left(S_{2}\right)u\left(y_{A2}\right),\\ V_{M}\left(n\right)=W\left(S_{1}\right)u\left(y_{M1}\right)+W\left(S_{2}\right)u\left(y_{M2}\right), \end{array}$$

where *u*(*y*_*Mj*_) and *u*(*y*_*Aj*_) are the utilities of the payoff, *W*(*S*_*j*_) is the subjective probability weight (attention given to event *S*_*j*_), and *n* is the *n*^*th*^ sample. Note that $\underset{j}{\sum }W\left(S_{j}\right)=1.$ Busemeyer and Townsend (1993) defined *P*(*n*) as the weighted preference state of choosing action automation *A* over manual *M*. They assumed that *P*(*n*) is determined by two factors: the previous state of preference *P*(*n* − 1) and the valence difference of *V*_*A*_(*n*) − *V*_*M*_(*n*). Thus, *P*(*n*) is defined as follows:


(6) $$\begin{array}{l} P\left(n\right)=\left(1-s\right)P\left(n-1\right)+\left[V_{A}\left(n\right)-V_{M}\left(n\right)\right]\\ \;=\left(1-s\right)P\left(n-1\right)+d+\epsilon \left(n\right), \end{array}$$

where *V*_*A*_(*n*) − *V*_*M*_(*n*) = *d* + ϵ(*n*) has an average of *d* and the related residual ϵ(*n*) represents the change in valence difference produced by the moment-to-moment fluctuations in attention during deliberation. The parameter *s* is the growth-decay rate, which determines the influence of the previous preference state *P*(*n* − 1). DFT offers an appropriate modeling approach to describe the decision to adopt automatic or manual control. The preference for automation over manual model (6) has been extended and applied in the modeling of trust and self-confidence in Gao and Lee (2006) and van Maanen and van Dongen (2005).

#### 2.1.4. Gao and Lee Model

Gao and Lee (2006) used Lee and See's (2004) definition of trust, a factor that influences decision-making. Gao and Lee (2006) first provided the formulation of the extended DFT model (EDFT) on the preference dynamics *P*(*n*),


(7) $$\begin{array}{l} P\left(n\right)=\left(1-s\right)P\left(n-1\right)+s\times d+\epsilon \left(n\right), \end{array}$$

which is essentially an autoregressive model that considers a linear combination of the previous preference state *P*(*n* − 1) and the new input on the current preference state *d* in an uncertain environment described by ϵ(*n*). The modeling approach of *P*(*n*) has been applied to develop a quantitative model of trust and self-confidence that is linked to decision-making in automation usage. The trust *T* and self-confidence SC take the following forms:


(8) $$\begin{array}{l} T\left(n\right)\;=\left(1-s\right)T\left(n-1\right)+s\times B_{CA}\left(n\right)+\epsilon \left(n\right),\\ \text{SC}\left(n\right)=\left(1-s\right)\text{SC}\left(n-1\right)+s\times B_{CM}\left(n\right)+\epsilon \left(n\right), \end{array}$$

where *B*_*CA*_ and *B*_*CM*_ are the input for the evolution of trust and self-confidence, representing that automation and manual control capabilities are the primary factors influencing the operator's decision to rely on automation or use manual control. The belief in the automation's capability (*B*_*CA*_) or the operator's manual capability (*B*_*CM*_) is constructed through a piece-wise function that uses the Fishbein and Ajzen (1975) framework, where beliefs represent an information base that determines attitudes, and attitudes determine intent and consequently, behaviors. Let ϵ_*P*_(*n*) be a random variable with zero mean and variance $\sigma_{P}^{2}$. The authors defined the preference of A over M *P*(*n*) as the difference between trust and self-confidence:


(9) $$\begin{array}{r} P\left(n\right)=T\left(n\right)-\text{SC}\left(n\right)=\left(1-s\right)P\left(n-1\right)+s\\ \times \left[B_{CA}\left(n\right)-B_{CM}\left(n\right)\right]+\epsilon_{P}\left(n\right), \end{array}$$

which characterizes multiple sequential decisions instead of the single decisions addressed by DFT. This model is based on psychological principles and depicts the dynamic interaction between the operator and automation. The dynamic interactions involve the relationship between the operator, the state of the automation, and the interface where the operators receive information. The model replicates empirical results on the inertia of trust and the non-linear relationship between trust, self-confidence, and reliance. The authors acknowledge two limitations to the model. First, it is assumed that the automation and operator capabilities are available, which are the primary input variables. Second, the fit obtained for the model validation is not enough, given that the fit must be done with a greater range of experimental data.

A useful feature of the basic DFT and extended DFT model is that it does not contain an explicit variable for risk, but it is considered through the SEU functions. Additionally, the model has the potential to be generalizable to other task environments involving human-automation interaction because the model considers the operator's preference based on their belief in the automation's capability and their manual capability, their trust, and self-confidence. Moreover, the model can be repeatedly updated with newly available information as the operator interacts with the automation.

#### 2.1.5. van Maanen et al. Model

van Maanen and van Dongen (2005) used the definition of trust from Falcone and Castelfranchi (2001) and referred to it as a mental state and belief of a cognitive agent *i* regarding the achievement of a desired goal through another agent *j* or through agent *i* itself. They implemented the framework of Decision Field Theory (DFT) to derive a model for task allocation where both humans and machines act together as a team. The model consists of four mathematical definitions: task execution state, trust state, allocation preference state, and preferred task execution states. The task execution and preferred task execution state are sequences of characters, while the trust state and allocation preference state are real numbers (∈ ℝ).

The authors considered that trust depends on past experiences and used the agent's task execution state to update the trust state. Furthermore, the model assumed that preferences are determined by trust in oneself and trust in others. Then, the allocation preference state was given by the difference of trust states corresponding to self-trust and trust in the machine. Finally, the previous states helped the operator make a preferred decision on the allocation task, which updated the preferred task execution state.

The authors proposed an experimental design to validate the model, where the goal was to predict the location of a disturbance that could only occur at one of three locations as a human-machine team. However, the authors did not clarify the payoff of a correct or incorrect task allocation, which is essential for the SEU (Subjective Expected Utility) functions in the DFT model. Hence, the assessment of risk in the model was not clearly defined. This model may not be generalizable for more complex environments where the operator faces more than three different environmental factors that can influence several different outcomes.

#### 2.1.6. Akash et al. Model

Akash et al. (2017) proposed a three-state model for trust in automation (*T*) by adopting the modeling work of Jonker and Treur (1999) and the concept of Hoff and Bashir (2015)'s classification of trust discussed in the introduction.

Jonker and Treur's (1999) trust model described the change in trust as proportional to the difference in experience and trust. Akash et al. (2017) adapted Jonker's model and introduced two additional states–Cumulative Trust (*C*_*T*_) and Expectation Bias (*B*_*X*_)–to accommodate the bias in human behavior due to perceptions of past trust and expectations as follows:


(10) $$\begin{array}{l} T\left(n+1\right)-T\left(n\right)=\alpha_{e}\left[E\left(n\right)-T\left(n\right)\right]+\alpha_{c}\left[C_{T}\left(n\right)-T\left(n\right)\right]\\ \;+\alpha_{b}\left[B_{X}\left(n\right)-T\left(n\right)\right],\\ C_{T}\left(n+1\right)\;=\left[1-\gamma \right]C_{T}\left(n\right)+\gamma T\left(n\right),\\ B_{X}\left(n+1\right)\;=B_{X}\left(n\right), \end{array}$$

where α_*e*_, α_*c*_, and α_*b*_ are called the experience rate factor, cumulative rate factor, and bias rate factor, respectively. Additionally, γ discounts older trust levels faster, and thus it can be called the trust discounting factor.

The specific assumptions of modeling trust in the model (10) are that the change in trust *T*(*n* + 1) − *T*(*n*) linearly depends on three terms: (a) the difference between experience and present trust *E*(*n*) − *T*(*n*), (b) the difference between cumulative trust and present trust *C*_*T*_(*n*) − *T*(*n*), and (c) the difference between expectation bias and present trust *B*_*X*_(*n*) − *T*(*n*). If the present experience is less than the present trust level, then the predicted trust level decreases and vice-versa.

The cumulative trust was defined as an exponentially weighted moving average of past trust levels to include the learned trust in the model using a weighted history of past trust levels. The expectation bias, which accounts for a human's expectation of a particular interaction with an autonomous system, is intended to be constant during an interaction, but it can change between different interactions.

Akash et al.'s (2017) model (10) is difficult to generalize for other scenarios because it is based on specifically asking participants whether they trust the automation or not. It assumes there is a way of measuring current levels of trust to predict future trust, so the prediction of trust based on real-time behaviors is not possible with this model.

### 2.2. Step Functions Approaches

Itoh and Tanaka (2000) define trust as the expectation or belief that automation is dependable. Their definition of trust evolves from the definition proposed by Rempel et al. (1985), which states that trust evolves from predictability, dependability, and faith. This modeling basis is consistent with the definition used in Muir (1994) and Muir and Moray (1996). Itoh and Tanaka (2000) proposed a step functional model for trust in automation by adopting the Rempel et al.'s (1985) definition. The authors define *X* as the universal set of all possible automation operating conditions, where *x, y* ∈ *X* and *x* < *y* means that *y* is more difficult than *x*. The trust model consists of three different sets: the faithful condition (*F*), the dependable condition (*D*), and the predictable condition (*P*). Additionally, the sets *UF*, *UD*, and *UP* refer to the complement of the faithful (*F*), dependable (*D*), and predictable condition (*P*), respectively. The Itoh-Tanaka model defines trust as a function of the automation's operating condition (*x*), which takes the following form:


(11) $$\begin{array}{l} t\left(x\right)=\{\begin{array}{ll} 1 & \text{if }x\in D\\ 0 & \text{if }x\in UD\;\text{or }x\in UF\\ \alpha_{x} & \text{if }x\in UP \end{array} \end{array}$$

Here, α_*x*_ ∈ [0, 1] depends on the operator's personality and/or experience with the automation. Several experimental studies on trust in automation measure trust using a 10-point rating scale, where a value of 1 represents *no trust in* and a value of 10 represents *complete trust*. However, this rating only provides a final measurement of trust and cannot give us a comprehensive understanding of its dynamics. The authors relate their model to this trust rating, where the trust rating is given by:


(12) $$\begin{array}{l} t_{s}=\frac{\int_{X}t\left(x\right)dx}{\left|X\right|}. \end{array}$$

This estimates the perceived trust value after the operator interacts with automation and is calculated by taking the mean of the function *t*(*x*). However, this model of trust does not assess task environment or risk as factors that influence the operator's trust. Additionally, this model is highly general to describe trust dynamics not only for automation but also for other beings or operational devices. Moreover, the authors use variables such as faith and the operator's personality but do not describe or suggest methodologies to measure these variables. As this model is described as a function of sets, it is not generalizable for use with other added variables such as risk, environmental factors, and the operator's task load.

#### 2.2.1. Monir et al. Model

Monir Rabby et al. (2020) define trust using a step function in which a person's level of trust in a robot depends on both the person's and the robot's performances. These two types of performances determine where the person is in the trust build-up process. Such regions were obtained from previous works (Rempel et al., 1985; Muir, 1989, 1994; Muir and Moray, 1996; Itoh and Tanaka, 2000) that suggest trust can be captured based on three aspects: predictability, dependability, and faith. The Monir Rabby et al. (2020) model is given by the following formula:


(13) $$\begin{array}{l} T\left(t\right)=\{\begin{array}{cc} 0 & ;\text{ for }R_{p}\left(t\right)<f_{P}\\ \epsilon & ;\text{ for }f_{P}\leq R_{p}\left(t\right)<f_{D}\\ min\left(1,\epsilon +tanh\left(c\Delta P\right)\right) & ;\text{ for }f_{D}\leq R_{p}\left(t\right)<f_{F}\\ 1 & ;\text{ for }R_{p}\left(t\right)\geq f_{F} \end{array} \end{array}$$

where *T* is the level of trust the person has in the robot, *t* is the time, *R*_*P*_ is the robot performance, Δ*P* = *R*_*P*_(*t*) − *f*_*D*_, *f*_*P*_ = σ*H*_*P*_(*t*), *f*_*D*_ = ρ*H*_*P*_(*t*), *f*_*F*_ is the robot performance at which trust reaches its maximum value, *H*_*P*_ is the person's performance, σ and ρ are small numbers, and *c* and ϵ are variables that depend on the person's preferences. In this model, the robot is in the unpredictable region when *R*_*P*_(*t*) < *f*_*P*_ as its performance is much lower than the person's performance, it is in the predictable region when *f*_*P*_ ≤ *R*_*P*_(*t*) < *f*_*D*_, it is in the dependable region after *R*_*P*_(*t*) ≥ *f*_*D*_ where the trust build-up process starts, and finally, it is in the faithful region when the trust value reaches its maximum at *R*_*p*_(*t*) = *f*_*F*_.

This model was proposed for the specific scenario of human-robot interaction in which a human supervises the correct execution of a classification task made by a robot. The experiment was conducted in a physical laboratory environment, and the task consisted of separating cubes of different colors by placing them on different counters. This model can be applied to other scenarios by modifying their definitions and measures. The separation of trust into different regions based on the performance of humans and robots is applicable to any other scenario of human-robot interaction. However, the way of measuring the robot and human performance may differ depending on the priority of the particular case of analysis. Furthermore, this model appears to assume some level of stability in the task environment, meaning that it may be limited in describing what happens with trust when anomalous factors in the environment generate situations affecting human or robot performance, specifically situations requiring trust repair.

### 2.3. Probability Approaches - Bayesian Network

#### 2.3.1. van Maanen et al. Model

van Maanen et al. (2007) used the Lee and See (2004) definition of trust, where trust is described as the attitude that an agent (human or automated aid) will help achieve an individual's goal in a situation characterized by uncertainty and vulnerability, and a cognitive state (Falcone and Castelfranchi, 2001). The van Maanen et al. (2007) model is based on the idea that when people work with automated decision aids, and vice versa, they perform better than when the person and aid work separately. Humans tend to overtrust or undertrust the aids or their own performance. Conversely, an automated aid can make quick and unbiased calculations on the operator's and their system performance based on previous successful and failed tasks. The model's objective is to verify the possibility that the aid can make more accurate trust assessments and accurate reliance decisions. Thus, the decision aid calculates its trust in the operator and in itself based on the reliance on decision-making capabilities each time there is feedback information.

In designing the decision aid, the authors derived a probabilistic model for trust using the binomial likelihood function, the Beta probability density function (PDF), and Bayes' rule to update estimations. Presuming the operator's and aid's behavior can be assessed as a "success" or "failure," then this can be described by the Bernoulli distribution. Because the Beta distribution is the conjugate prior to the Bernoulli distribution in Bayesian inference, this saves numerical computation for the posterior in Bayesian inference. Hence, the aid uses Bayes' rule to update its estimations over the different capability values that the operator or aid can have. The aid needs to estimate the probability of a successful outcome in each trial, $\theta_{a}^{x}$ where *x* ∈ {prediction, reliance} and *a* ∈ {operator, aid}.


(14) $$\begin{array}{l} p\left(\theta |n,N,r,s\right)\propto \left[\underset{\text{Likelihood}}{\underset{︸}{\theta^{k}{\left(1-\theta \right)}^{N-n}}}\right]\left[\underset{\text{prior}}{\underset{︸}{\theta^{r-1}{\left(1-\theta \right)}^{s-1}}}\right]\\ \;\propto \theta^{n+r-1}{\left(1-\theta \right)}^{N-n+s-1} \end{array}$$

To capture the trust dynamics, participants were asked to perform a pattern recognition task with the help of a decision aid. The operators needed to maximize the number of correct answers by relying on their own predictions or the aid's predictions for pattern recognition. Thus, the factors considered in the model are the number of successes and failures for the person and the aid, which refer to the number of correct and incorrect responses each made. Additionally, the task environment and model do not assess a risk variable. Even though the model could be modified to calculate the person's trust assessment, the model is not generalizable for other human-automation interactions in more complex environments. However, this model idea could be incorporated as part of a larger model that enables us to predict trust and reliance with automation in a high cognitive task with a complex environment.

#### 2.3.2. Xu and Dudek Model–Dynamic Bayesian Network

Xu and Dudek (2015) introduced their own definition of *trust*, which they defined as a person's belief in the competence and reliability of another. They suggested that the level of reliance indicates the level of trust. To explore this concept further, they developed a Dynamic Bayesian Network model for a person's level of trust in a robot teammate. The model assumes that the person occasionally intervenes to aid the robot in completing a series of tasks, acting as a "supervisor" of the robot.

The probabilistic model developed by Xu and Dudek (2015), is called the Online Probabilistic Trust Inference Model (OPTIMo). It formulates a Bayesian beliefs network over the person's moment-to-moment trust states. The Bayesian model accommodates an arbitrary categorical belief for initial trust and incorporates variable-rate sources of information in a probabilistic manner.

The model relates the operator's latent trust state (*t*_*k*_) to the robot's task performance (*p*_*k*_ ∈ [0, 1]), uses human interventions (*i*_*k*_ ∈ {0, 1}), trust change reports (*c*_*k*_ ∈ {−1, 0, +1, ∅}), absolute trust feedback (*f*_*k*_ ∈ {[0, 1], ∅}), and *e*_*k*_ the presence of a task change for a time window *k*. The model employs a set of equations, including:


(15) $$\begin{array}{l} \underset{\text{Expected Update of Trust}}{\underset{︸}{P\left(t_{k},t_{k-1},p_{k},p_{k-1}\right)}}\approx N(t_{k};t_{k-1}+\omega_{tb}+\omega_{tp}p_{k}\\ \;+\omega_{td}\left(p_{k}-p_{k-1}\right),\sigma_{t}), \end{array}$$

(16) $$\begin{array}{l} \underset{\text{Probability of Interventions}}{\underset{︸}{O\left(t_{k}=1,t_{k-1},i_{k},e_{k}\right)}}:=S\left(\omega_{ib}+\omega_{it}t_{k}+\omega_{id}\Delta t_{k}+\omega_{ie}e_{k}\right), \end{array}$$

(17) $$\begin{array}{l} \underset{\text{Change of Trust}}{\underset{︸}{O_{c}\left(t_{k},t_{k-1},c_{k}\right)}}:=Prob\left(c_{k}|t_{k},t_{k=1}\right), \end{array}$$

(18) $$\begin{array}{l} Prob\left(c_{k}=+1|t_{k},t_{k=1}\right)=\beta_{c}+\left(1-3\beta_{c}\right)\cdot S\left(\kappa_{c}\left[\Delta t_{k}-o_{c}\right]\right),\\ Prob\left(c_{k}=-1|t_{k},t_{k=1}\right)=\beta_{c}+\left(1-3\beta_{c}\right)\cdot S\left(\kappa_{c}\left[-\Delta t_{k}-o_{c}\right]\right),\\ Prob\left(c_{k}=0|t_{k},t_{k=1}\right)=\underset{\text{User's Absolute Trust}}{\underset{︸}{O_{f}\left(t_{k},f_{k}\right)}}\\ \;:=Prob\left(f_{k}|t_{k}\right)\approx N\left(f_{k};t_{k},\sigma_{f}\right). \end{array}$$

In the equations above, N(x;μ,σ) denotes a Gaussian distribution. Parameters such as ω_*tb*_, ω_*tp*_ and ω_*td*_ reflect bias, current task performance, and difference in robot's performance, respectively, of the operator's updates. The sigmoid function S(x) is also used, and parameters such as ω_*ib*_, ω_*it*_, ω_*id*_ and ω_*ie*_ quantify bias and weights for an intervention *i*_*k*_. Additionally, β_*c*_ captures users' self-reported erroneous trust changes.

The model was validated using data from a laboratory-simulated experiment, and the authors accurately predicted human trust-induced behaviors. However, neither the model nor the experiment used for model validation took risk into account as a factor. This model differs from others because it uses the operator's reliance behavior on automation to infer the level of the operator's trust. This framework has the potential to model trust dynamics for other human-automation interaction task scenarios that involve an operator's reliance behaviors throughout a trial. Additionally, other variables could be added to the model, such as weights for risk and difficulty of the changing elements in the task, as well as an expected update of the operator's self-confidence to complete the task manually. Lastly, the model can be applied to other types of automation.

#### 2.3.3. Guo and Yang Model

Guo and Yang (2021) used the definition of trust given by Lee and See (2004) and acknowledged that trust in automation is dynamic and can increase or decay as the human interacts with the automation. To do so, they used a Beta distribution to develop a personalized trust prediction model and applied Bayesian inference to calculate the Beta distribution parameters. The authors adhered to the following assumptions in their model derivation.

1. Trust at time *i* is influenced by trust at time *i* − 1.
2. Trust is more heavily impacted by negative experiences with the automation compared to positive experiences.
3. As humans engage with an automation multiple times, their trust in the agent stabilizes.

This study postulates that following the automation's completion of the *i*th task, the human's temporal trust *t*_*i*_ follows a Beta distribution with parameters α_*i*_ and β_*i*_. These parameters are updated by,


$$\begin{array}{l} \alpha_{i}=\{\begin{array}{ll} \alpha_{i-1}+w_{s}, & \text{if }p_{i}=1\\ \alpha_{i-1}, & \text{if }p_{i}=0 \end{array}\\ \beta_{i}=\{\begin{array}{ll} \beta_{i-1}+w_{f}, & \text{if }p_{i}=1\\ \beta_{i-1}, & \text{if }p_{i}=0 \end{array}, \end{array}$$

where *p*_*i*_ ∈ {0, 1} is the automation's performance on the *i*th task, which takes a value of one (*p*_*i*_ = 1) to indicate a success and a value of zero (*p*_*i*_ = 0) to indicate a failure. Additionally, *w*_*s*_ and *w*_*f*_ represent the gains due to positive and negative experiences with the automation. Therefore, the predicted trust (${\hat{t}}_{i}$) at the completion of the *i*th task can be estimated by the mean of *t*_*i*_. Since *t*_*i*_ ~ *Beta*(α_*i*_, β_*i*_), then ${\hat{t}}_{i}$ is given by,


(19) $$\begin{array}{l} {\hat{t}}_{i}=E\left(t_{i}\right)=\frac{\alpha_{i}}{\alpha_{i}+\beta_{i}} \end{array}$$

The model was validated using data from Yang et al. (2017) and compared with Lee and Moray's (1992) and Xu and Dudek's (2015) models. The model proposed by Guo and Yang (2021) outperformed the two existing trust prediction models. The authors acknowledge the following four limitations: (1) the model assumes that the automation's ability remains constant across all interactions; (2) the parameters used in the model are assumed to be independent of each other; (3) the model assumes that the automation's performance is either good or bad and is immediately available after a task; and (4) each participant in the experiment had 100 interaction episodes with the automation in a relatively short period of time. Since the model utilizes the automation's performance and reliability, along with the human's self-reported trust history, it possesses the potential to be generalized for other human-automation task scenarios that record alterations in these measures. Furthermore, the model has the potential to include other factors that influence trust in automation and can be enhanced with behavioral data gathered during the human-automation interaction.

## 3. A Unified Mathematical Framework

Having discussed the current state of research on trust in automation and the various models proposed to study it, we propose a unified mathematical framework to study trust in automation. In particular, we identify some key areas where further research is needed to advance our understanding of human-automation trust and its applications.

Researchers have made significant headway in identifying and understanding factors that influence trust in automation and the relationships between those factors through experiments and mathematical modeling approaches. In a seminal review on this topic, Lee and See (2004) emphasized the increasing need to understand how people trust complex automated systems. However, the utility of trust – and its role in affecting behaviors and human-automation performance – becomes limited when it is operationalized as a static outcome, as is often the case in many experimental studies. Instead, to more closely align with trust theory, trust should be operationalized as part of an interactive and dynamic process with multiple sources of influence and at various timescales (Lee and See, 2004; Hoff and Bashir, 2015; Chiou and Lee, 2021).

### 3.1. Time Scales of Mathematical Models

Mathematical models can describe and connect different dynamical components or variables that operate at different timescales. These components or variables can be deployed, tested, and measured through observational experiments. When dynamical processes have different timescales, dynamical models allow us to more deeply explore the relations among the measurable components captured and understand which of these has more weight at different times within the domain of study.

All mathematical models reviewed in this paper support the idea that *trust in automation* is shaped by complicated dynamical processes involving the human operator, automated system, and environment, all of which intertwine and impact each other over time. One major limitation of current models of trust is that they do not adequately explain the relationship between trust measurements and the overall model or its individual components. Additionally, some of these models are derived without using rigorously defined components, making them challenging to measure through data collection methods. For example, Itoh and Tanaka's (2000), Muir's (1994), and Muir and Moray's (1996) models include components such as faith, predictability, and dependability, but these components are not clearly defined or measurable during the model development process. Furthermore, current models have gaps in describing the intricate dynamics of trust in automation as a function of one or more of the following interactions: automation's capabilities and performance, the operator's experiences and performance, and the environment in which such interactions occur. Hence, there is a need for models that incorporate the aforementioned elements with precise definitions and methods for their quantification.

This motivates us to develop a unified modeling framework that defines essential components for trust that are measurable through experimental trials and/or surveys. To achieve this, we start with situational, learned, and dispositional trust definitions in Hoff and Bashir (2015), which can be classified into the following three timescales:

1. **Short-timescale (i.e., situational; e.g., in seconds/minutes):** decision making that is related to predictability and the present situation such as risk,
2. **Intermediate-timescale (i.e., learned; e.g., in hours/days):** reliance and performance information related to dependability and learned trust from previous experience, and
3. **Long-timescale (i.e., dispositional; e.g., in years/decades):** linked to dispositional trust developed from consistent messaging or socialization, including culture.

As previously discussed in the introduction, Hoff and Bashir (2015) identified dispositional, situational, and learned trust and the specific components that fall under each category. However, a timescale classification becomes helpful to understand the timescale of these sources of trust when designing experiments and selecting data collection methods. One distinction between Hoff and Bashir's framework and this timescale classification is that some components of situational trust described in Hoff and Bashir (2015) can also occur during the interaction with automation and be classified as short-timescale. Secondly, Hoff and Bashir's classification falls short in offering clear methods for quantifying each component and in describing the exact nature of the relationship between these components and trust. Our timescale classification offers precise definitions and methods for the quantification of each component, which is particularly helpful when designing experiments and selecting data collection methods.

The models reviewed in this article incorporate one or two, or all of these timescales in the dynamic trust process. For example, the Lee and Moray (1992, 1994) model studies intermediate timescale processes because it considers the automation's performance and occurrence of faults in trials recorded over 3 days. The Muir (1994) and Muir and Moray (1996) models fall into the long timescale because they consider either the persistence of natural laws or faith, which tend to involve a person's experiences over several years. The Akash et al. (2017) model studies short and long timescale processes as it considers learned trust, which is dynamically influenced by the system's performance and people's past experiences based on their self-reported demographic information. The Itoh and Tanaka (2000) model considers faith and two other conditions based on the automation's performance. Then the model studies intermediate and long timescale processes. Models constructed through Decision Field Theory, such as Busemeyer and Townsend (1993), Gao and Lee (2006), and van Maanen and van Dongen (2005), model processes within all three timescales because the models are linked to decision-making, reliance on automation (short-timescale), performance (intermediate-timescale), and belief (long-timescale). The Xu and Dudek (2015) model has both short and intermediate timescale components as it accounts for the automation's performance and the human decision-making to calculate what the authors refer to as a trust state.

Models of trust dynamics in the literature reflect the need to incorporate multiple timescales since trust dynamics involve complicated processes that interact with each other at different timescales. To validate and improve trust models, we must include components and variables that are measurable and can be evaluated through experimental trials of human-automation interaction. For example, the Monir Rabby et al. (2020) model uses the strength of prior beliefs in the automation's success or failure, which belongs to the intermediate or long timescale process. However, the authors used a dataset in which participants' trials were separated by seconds or minutes. This type of mismatch prevents us from having a better understanding of trust dynamics and originates from the components of the proposed model that are not defined in the correct timescales and cannot be measured by experimental trials.

To mathematically study the human-automation interaction and trust in automation, it is important to identify the essential components/elements that are measurable, at what timescales these elements take place, how those elements of the interaction happen, and how these are influenced by previous experiences. For example, as an operator uses automation, they will observe and remember its capabilities, performance, and reliability. Therefore, we cannot assume that the observable reliance on automation at some point is independent of all the previous interactions since the operator started using said automation. Furthermore, as people interact with automation to accomplish complex cognitive tasks, unforeseen events may limit the time they have to gather information and make decisions.

A mathematical model of trust in automation can capture various processes that occur before, during, and after in-the-moment interactions. By including short-timescale processes, we can capture these immediate interactions. Adding intermediate-timescale processes can help us observe how reliance changes as the operator learns the automation's capabilities and performance under different situations that may occur. Lastly, incorporating long-timescale processes can help us address individual variability in reliance behavior due to ingrained preferences, beliefs, social group relationships, and culture. We can use existing methods and conceptualized measurements (Kohn et al., 2021) to describe these processes and expand their definitions if necessary.

For instance, long-timescale factors such as personality, general experiences, impressions, and beliefs can be measured using surveys with scaled responses. We can use patterns of observed behaviors and related session-level measures of decision performance to capture intermediate timescale processes. For short-timescale measurements, we can include workload, reaction times for off-nominal events, perceived risk within specific decision contexts, and moment-to-moment decisions (e.g., deciding to continue engaging the automation or switching to manual control). While survey and observational methods can be applied across all timescales, we are not suggesting that these methods, more broadly speaking, are mutually exclusive across the timescales. However, *what* perception or behavior you are measuring exactly (rather than *how* you are measuring it) is more the point of these categories.

Mathematical models of trust in automation that incorporate multiple timescales can become more useful as they capture processes at different levels/timescales of study, which makes them capable of connecting and creating feedback between these timescales. This allows us to analyze mathematically how different elements (e.g., parameters) in each process affect not only the process they belong to but also other processes at different levels/timescales of study.

### 3.2. Trust in Automation Framework

It is natural to ask how to model the dynamic processes of trust using these three timescales. By considering where mathematical models fall within the three timescales, we propose that we can better capture yet-identified factors, patterns, and relationships that impact human trust during interactions with automated systems or devices. With this proposal in mind, we propose a unified framework to model trust in automation, as well as the derivation of the dynamic measurements used by the mathematical model. The mathematical derivation is done for a particular human-automation work structure where the automation could be engaged and disengaged at the operator's discretion to receive aid in completing tasks. Furthermore, the model is aimed at a task environment that tracks the automation's and human's performance, as well as the operator's reliance throughout the interaction (see Drnec and Metcalfe, 2016; Gremillion et al., 2016). We aim to develop a practical model capable of accurately predicting and depicting the dynamics of trust.

It is known that trust influences decision-making and, therefore, reliance on automation. Observing reliance on automation suggests that the operator trusts the automation at some time *t* to perform a set of tasks. Conversely, observing lower reliance implies that the operator may not trust the automation to successfully perform a set of tasks. Then, what factors influence decision-making? We can use risk, performance, workload, and environment, as found by Kohn et al. (2021). These findings are needed to derive the mathematical model of trust in automation on the short- and intermediate-timescale. Recall that we classified the processes of decision-making and risk as short-timescale and the processes of reliance and performance as intermediate-timescale. So, how would we model the decision-making process that occurs on a short timescale? We need to determine values of risk, performance, workload, and unforeseeable consequences due to the stochastic nature of the environment. We start the model derivation by defining measurable variables for each of the necessary factors that influence trust in automation, such as relative risk (belonging to a short timescale) and reliance (belonging to the intermediate timescale).

#### 3.2.1. Measurable Factors of Trust

Having defined trust as a crucial factor in human-automation interaction, we can now identify its measurable components, one of which is risk and reliance. In this subsection, we delve into the concept of risk, relative risk, reliance, and relative reliance and propose measures to quantify it in the context of automation usage and manual control.

##### 3.2.1.1. Risk

According to Sitkin and Pablo (1992), risk is "the extent to which there is uncertainty about whether potentially significant and/or disappointing outcomes of decisions will be realized." Thus, we can define the risk of automation usage over time [*t*_0_, *t*] as follows:


$$\begin{array}{l} R_{a}\left(t\right)=\frac{\begin{array}{l} \text{measurements of failed tasks}\\ \;\text{during automation on over time }\left[t_{0},t\right] \end{array}}{\begin{array}{l} \text{measurements of all tasks}\\ \;\text{during automation on over time }\left[t_{0},t\right] \end{array}} \end{array}$$

Similarly, we can define the risk of manual control over time [*t*_0_, *t*] as follows:


$$\begin{array}{l} R_{m}\left(t\right)=\frac{\begin{array}{l} \text{measurements of failed tasks}\\ \;\text{during manual control over time }\left[t_{0},t\right] \end{array}}{\begin{array}{l} \text{measurements of all tasks}\\ \;\text{during manual control over time }\left[t_{0},t\right] \end{array}} \end{array}$$

These definitions allow us to define risk using automation at varied timescales depending on the length of *t*_0_ − *t*. Based on the measurements (e.g., varied score systems), these expressions allow for individual assessment of the risk of automation usage or manual control, decision-making, and perception of the level of risk in a constantly changing environment. To compare the level of risk between automation usage and manual control, we can use the *Relative Risk* of using automation to manual over time [*t*_0_, *t*], which is denoted by $RR\left(t\right)=\frac{R_{a}\left(t\right)}{R_{m}\left(t\right)}$. These measurements are applicable to any study tracking the operator's and automation's performance across the trials through scoring systems.

For example, if the study does not include a reward system for completing tasks (see the work of Drnec and Metcalfe, 2016; Gremillion et al., 2016), relative risk measurement can be modified to only include task violation penalties. This measure of association can be interpreted in three different ways. If *RR*(*t*) < 1, then there is a decreased risk of task violations while using the automation, and therefore the operator could be predisposed to engage the automation. If *RR*(*t*) > 1, there is an increased risk of task violations while using the automation, and therefore the operator should refrain or be careful to engage the automation. Lastly, if *RR*(*t*) = 1, then both engaging the automation and using manual control have the same amount of risk, and, therefore, the operator can choose any of the two options. The relative risk measurement presents an opportunity to deeply study human behavior and decision-making while performing a highly demanding cognitive task. The variables used for its computation can be updated in real-time as the environment changes during a task. The resulting dynamics allow researchers to study relative risk and human perception of risk throughout each trial. Refer to Rodriguez Rodriguez et al. (2022) for an example of the application of this measurement.

##### 3.2.1.2. Reliance

Reliance on automation is defined as "the fraction of time that human operators have the automation engaged" (Lee and Moray, 1992). In previous studies, reliance has been measured as a finite constant describing the total percentage of reliance the operator had for any given trial. Instead, we propose this measurement as a time-dependent variable. Similar to the measurement of risk and relative risk, the reliance measurement can be updated in real time as the operator chooses to engage and disengage the automation in a changing environment.

To define the reliance measurement, assume that a human operator is performing a task over some period of time *T*. Over the time interval [0, *t*] ⊂ [0, *T*] where *t* ≥ 0, assume there are *K* time sub-intervals in which the operator chooses to engage the automation, i.e., [*t*_1_, *t*_2_], [*t*_3_, *t*_4_], ..., [*t*_2*K*−1_, *t*_2*K*_] where *t*_2*K*_ ≤ *t*. Then the reliance of the operator at time *t* ∈ [0, *T*] is defined as:


(20) $$\begin{array}{l} RIL\left(t\right)=\frac{1}{t}\overset{K}{\underset{j=1}{\sum }}\left(t_{{}_{2j}}-t_{{}_{2j-1}}\right) \end{array}$$

This definition allows researchers to know what proportion of time the automation has been used by the operator at any time *t* during the whole trial, which lasts some time *T*. Thus, the measurement of reliance will always have a value between zero and one, *RIL*(*t*) ∈ [0, 1], for any time *t* in *T*.

Additionally, reliance can be measured in a shorter time scale by measuring the percentage of time the automation was used since it was engaged the previous time. Recall that there are *K* time sub-intervals in which the operator chooses to engage with the automation. If the automation is engaged at time *t* (e.g., *t* ∈ [*t*_2*j*−1_, *t*_2*j*_] for some positive integer 1 ≤ *j* ≤ *K*), then we define Instant Reliance at time *t* as *IRIL*(*t*) = 1. Otherwise, if *t* ∈ [*t*_2*j*_, *t*_2*j*+1_] for some positive integer 1 ≤ *j* ≤ *K* − 1, then Instant Reliance is defined as:


(21) $$\begin{array}{l} IRIL\left(t\right)=\frac{t_{{}_{2j}}-t_{{}_{2j-1}}}{t-t_{{}_{2j-1}}}<1 \end{array}$$

This measurement allows us to observe when the automation was engaged and how reliance starts to decrease the moment the human operators change to manual control. Measuring reliance through time allows us to observe the operators' learning time frame of the automation's performance and capabilities and the operators' shifts of reliance with different types of automation and specific environmental changes. Refer to Bustamante Orellana et al. (2022) for an example of the application of this measurement.

### 3.3. An Application of the Modeling Framework

Building on the discussion of trust in human-automation interaction in the previous section, we can now explore an example of a modeling framework that can be used to model trust with short and intermediate timescales. Through the measurements we have discussed, we can provide an example of modeling trust in automation with short and intermediate timescales based on available data of people completing a leader-follower task in a simulated environment with the assistance of an automated driving system (Drnec and Metcalfe, 2016; Gremillion et al., 2016). Adopting the modeling approaches of Muir (1994), Muir and Moray (1996), and Busemeyer and Townsend (1993), we can propose the mathematical formulation of trust as follows:


(22) $$\begin{array}{l} Tr\left(t\right)=Tr_{M}\left(t\right)+Tr_{S}\left(t\right)+\text{Environment} \end{array}$$

An example of the intermediate timescale of modeling trust in automation *Tr*_*M*_(*t*) at time *t* can be modeled as a function of the average relative risk $\frac{\overset{K-1}{\underset{i=1}{\sum }}RR\left(t_{i}\right)}{K-1}$ (where *t*_*i*_ and *K* were defined for Equation 20), the reliance *RIL*(*t*), and the automation's performance reflected by:


(23) $$\begin{array}{l} p\left(t\right)=\frac{\begin{array}{l} \text{lost points due to failed tasks}\\ \;\text{when using automation over }\left[t_{0},t\right] \end{array}}{\text{total lost points over }\left[t_{0},t\right]} \end{array}$$

The larger the value of *p*(*t*), the worse the performance, and the smaller the value of *p*(*t*), the better the performance. Thus, an example of *Tr*_*M*_(*t*) could be expressed as follows:


$$\begin{array}{l} Tr_{M}\left(t\right)=\frac{\sum_{i=1}^{K-1}RR\left(t_{i}\right)}{K-1}\times RIL\left(t\right)\times p\left(t\right) \end{array}$$

This suggests that the operator has a large value of trust in automation if (1) the reliance *RIL*(*t*) is big, (2) the average relative risk of using automation is high, and (3) the automation's performance is bad. On the contrary, the operator has little trust in automation if the reliance *RIL*(*t*) is small, the average relative risk of using automation is low, and the performance is good.

An example of the trust dynamics in a short timescale can be modeled as a function of the relative risk over the small time interval of Δ*t* and instant reliance values at time *t*. Hence, the short timescale trust value at time *t* can be given by:


$$\begin{array}{l} Tr_{S}\left(t\right)=RR\left(\Delta t\right)\times IRIL\left(t\right) \end{array}$$

The proposed model allows us to include behavioral and environmental factors through reliance on automation and the joint human-autonomy team performance in a changing environment. This example of modeling trust in automation only considers behavior data with intermediate and short timescales. Additionally, this example is specific to a particular human-automation work structure such as the operator having the freedom to choose when to hand-off partial or complete control to the automation for the completion of a set of tasks. It is possible that we could make the short timescale expression of the model more reliable by implementing real-time data such as physiological data.

## 4. Discussion

Toward a more reliable and responsive model, there is potential for including sensor-based physiological measures. Usually, survey measures are a one-time event; however, some studies administer questionnaires multiple times during a task to capture changes in trust over time (Cummings et al., 2021). Yet, no matter how often survey measures are administered, they cannot always capture a person's in-the-moment responses to unforeseen perturbations, workload, and perceived changes in performance (Huang et al., 2020). Although current efforts in this area remain limited, sensor-based physiological measures like pupil dilation (Aygun et al., 2022), electroencephalography (EEG), and Galvanic skin response (GSR) could allow us to more closely monitor and respond to trust indicators, and triangulate those data with the task contexts and other well-established trust measures like associated behaviors (e.g., reliance data, perceived risk, and changes in decisions) to detect weak points of a human-automation team in real-time (DeCostanza et al., 2018) and paint a fuller picture of trust dynamics in specific situations and across patterns of situations over time. In some task environments, interaction or communication data between operators and automation could also provide a stream of real-time indicators (Lee and Kolodge, 2020). Recent work by Kohn et al. (2021) reviews the many methods that have been used to successfully measure and assess trust in automation.

Since physiological and behavioral time-series datasets are typically large and complex, there is value in utilizing both machine learning and dynamical modeling approaches to interpret the data. These approaches, along with recent advancements in sensors and computing, have the potential to provide new insights into trust in automation and can aid in the development of powerful mathematical models. However, understanding the inner workings of machine learning models and their prediction mechanisms can be challenging (Ribeiro et al., 2016). This can be an issue when model interpretability is critical, as is often the case in high-accountability environments. Fortunately, more interpretable models such as decision trees, linear models, and rule-based models are available (Molnar et al., 2020) as are techniques to make more complex models more interpretable (Breiman, 2001; Liaw and Wiener, 2007).

The combination of real-time data streams and computational analysis approaches offers a source of analytical insights that avoids the interruption of tasks to solicit survey responses from participants. To the extent that these data hold promising indicators of trust, their analysis can help identify critical factors and relationships that are predictive of trust-related behaviors within those data streams. The factors and relationships identified through machine learning and computational analysis can then be included in a mathematical model of trust. Our ongoing research project consists of expanding the framework for a mathematical model of trust to include dynamical measures of workload and account for the stochasticity of a changing environment. We aim to establish the foundation of these powerful mathematical models by combining dynamic systems and machine learning components. The advantages of these models are that they permit rigorous mathematical analyses and validation with various human-automation interaction settings, thus providing the research community with a powerful tool to study trust dynamics.

## Conclusion

In this review, we have gathered and summarized prominent mathematical models of trust in automation derived from the human factors literature and the concept of trust in automation that has evolved since the nineties. Although researchers have made significant progress in identifying the complex dynamic processes involved in trust, there is a need to map how these processes may interact with each other and become a function of trust. To this end, we have introduced a three-timescale classification of these models and the processes they describe. Because trust is a dynamic process, rigorous definitions of reliance, risk, and performance are also defined as dynamic measures. We have described an example of a two-timescale model based on the proposed framework to show how processes that take place in the same timescale, such as performance and reliance, can both contribute to evolving trust. However, the model has some limitations, such as excluding the third long-timescale and not including an appropriate mathematical expression that considers how the environment can affect the operator. The research community continues to face the challenge of formulating better data collection methods that would enable better derivation of trust indicators and thus better measurement of trust. We hope that our effort serves as a toolbox for different model formulations previously used by the scientific community and helps researchers continue to improve trust modeling through our modeling framework.

## Author contributions

LR, CB, EC, and YK contributed to the conceptualization of the study and wrote the first draft of the manuscript. LR, CB, and YK contributed on the formal analysis of the models. EC and YK provided supervision. All authors contributed to manuscript revision, read, and approved the submitted version.

## Author disclaimer

The views and conclusions contained in this document are those of the authors and should not be interpreted as representing the official policies, either expressed or implied, of the Army Research Office or the U.S. Government. The U.S. Government is authorized to reproduce and distribute reprints for Government purposes notwithstanding any copyright notation herein.
